# The millipede genus *Eviulisoma* Silvestri, 1910 in Kenya, with descriptions of new species (Diplopoda, Polydesmida, Paradoxosomatidae)

**DOI:** 10.3897/zookeys.459.8621

**Published:** 2014-12-01

**Authors:** Didier VandenSpiegel, Sergei I. Golovatch

**Affiliations:** 1 Musée Royal de l’Afrique centrale, B-3080 Tervuren, Belgium; 2 Institute for Problems of Ecology & Evolution, Russian Academy of Sciences, Leninsky pr. 33, Moscow 119071, Russia

**Keywords:** Diplopoda, *Eviulisoma*, taxonomy, new species, key

## Abstract

The genus *Eviulisoma*, the largest among Afrotropical Paradoxosomatidae, currently encompasses 36 species or subspecies, including six new from Kenya: *Eviulisoma ngaia* **sp. n.**, *Eviulisoma ngaiaorum* **sp. n.**, *Eviulisoma taitaorum* **sp. n.**, *Eviulisoma taita* **sp. n.**, *Eviulisoma kirimeri* **sp. n.** and *Eviulisoma kakamega* **sp. n.** In addition, *Eviulisoma alluaudi* Brolemann, 1920 and *Eviulisoma silvestre* (Carl, 1909) are recorded for the first time beyond their type localities in Kenya and Tanzania, respectively, based on new material from Kenya. A key is given to all ten species of the genus presently reported from Kenya.

## Introduction

The genus *Eviulisoma* Silvestri, 1910 is the largest among Afrotropical Paradoxosomatidae, currently known to encompass 30 species or subspecies in central and eastern Africa (Nguyen and Sierwald 2013). The reader is referred to Jeekel (2003) for a most useful review of taxonomic research into *Eviulisoma*, a detailed new diagnosis, an outline of informal species groups and a key to most of the constituent species.

The following checklist of *Eviulisoma* species or subspecies has been extracted from Jeekel (2003) and Nguyen and Sierwald (2013):

1. *Eviulisoma cavallii* (Silvestri, 1907), the type species, from Uganda and Rwanda;

2. *Eviulisoma alluaudi* Brolemann, 1920, from Kenya;

3. *Eviulisoma boranicum* Manfredi, 1939, from Ethiopia;

4. *Eviulisoma castaneum* Attems, 1953, from the Democratic Republic of the Congo;

5. *Eviulisoma cervicorne* (Attems, 1927), from an unknown locality in Africa;

6. *Eviulisoma congicolens* (Chamberlin, 1927), from the Democratic Republic of the Congo;

7. *Eviulisoma cylindricum* Attems, 1953, from the Democratic Republic of the Congo;

8. *Eviulisoma cylindricum simile* Attems, 1953, from the Democratic Republic of the Congo;

9. *Eviulisoma dabagaense* Kraus, 1958, from Tanzania;

10. *Eviulisoma debile* Attems, 1938, from the Democratic Republic of the Congo;

11. *Eviulisoma egregium* Attems, 1938, from the Democratic Republic of the Congo;

12. *Eviulisoma fossiger* (Carl, 1909), from Tanzania;

13. *Eviulisoma graueri* Attems, 1944, from the Democratic Republic of the Congo;

14. *Eviulisoma insulare* Brolemann, 1920, from Zanzibar Island, Tanzania;

15. *Eviulisoma iugans* (Chamberlin, 1927), from the Democratic Republic of the Congo;

16. *Eviulisoma iuloideum* (Verhoeff, 1941), from Tanzania;

17. *Eviulisoma jeanneli* Brolemann, 1920, from Kenya;

18. *Eviulisoma kwabuniense* Kraus, 1958, from Tanzania;

19. *Eviulisoma lanceolatum* Attems, 1953, from the Democratic Republic of the Congo;

20. *Eviulisoma muturanum* Attems, 1937, from both the Democratic Republic of the Congo and the Republic of the Congo (Brazzaville);

21. *Eviulisoma obesum* Attems, 1953, from the Democratic Republic of the Congo;

22. *Eviulisoma obscurum* Attems, 1937, from the Democratic Republic of the Congo;

23. *Eviulisoma pallidum* Attems, 1939, from Kenya;

24. *Eviulisoma schoutedeni* (Attems, 1929), from the Democratic Republic of the Congo;

25. *Eviulisoma silvaticum* Attems, 1953, from Rwanda;

26. *Eviulisoma silvestre* (Carl, 1909), from Tanzania;

27. *Eviulisoma somaliense* Ceuca, 1971, from Somalia;

28. *Eviulisoma tertalinus* Manfredi, 1941, from Ethiopia;

29. *Eviulisoma tritonium* Attems, 1937, from the Democratic Republic of the Congo;

30. *Eviulisoma ussuwiense* (Carl, 1909), from Tanzania.

Prompted by the discovery of several new or poorly-known congeners in Kenya, eastern Africa, this paper focuses on their descriptions or records, as well as presenting a key to all *Eviulisoma* species currently known to occur in Kenya.

## Material and methods

The material underlying the present contribution was taken in Kenya in 1999–2004. Most of the types are housed in the collection of the Royal Museum for Central Africa, Tervuren, Belgium (MRAC), a few paratypes have been donated to the Zoological Museum, Moscow State University, Moscow, Russia (ZMUM + entry number).

SEM micrographs were taken using a JEOL JSM-6480LV scanning electron microscope. After examination, SEM material was removed from stubs and returned to alcohol, all such samples being kept in MRAC.

Line drawings were very skillfully executed by Mrs Nadine Van Noppen (MRAC).

## Results

### Eviulisoma ngaia sp. n.

Taxon classificationAnimaliaPolydesmidaParadoxosomatidae

http://zoobank.org/F10DDEC4-2738-42E8-833A-A08B839D5DFB

Fig. 1 , Map 1

#### Type material.

Holotype ♂ (MRAC 20799), Kenya, Ngaia Forest, N00°19', E38°02', ca 1070 m a.s.l., 2.XII.2002, leg. D. VandenSpiegel.

Paratypes: 3 ♂, 1 ♀, 1 juv. (MRAC 22634), 1 ♂ (ZMUM ρ2442), same data, together with holotype; 1 ♂ (MRAC 20703), same data, 3.XII.2002, leg. D. VandenSpiegel.

#### Name.

To emphasize the type locality, a noun in apposition.

#### Diagnosis.

**Figure 1.**
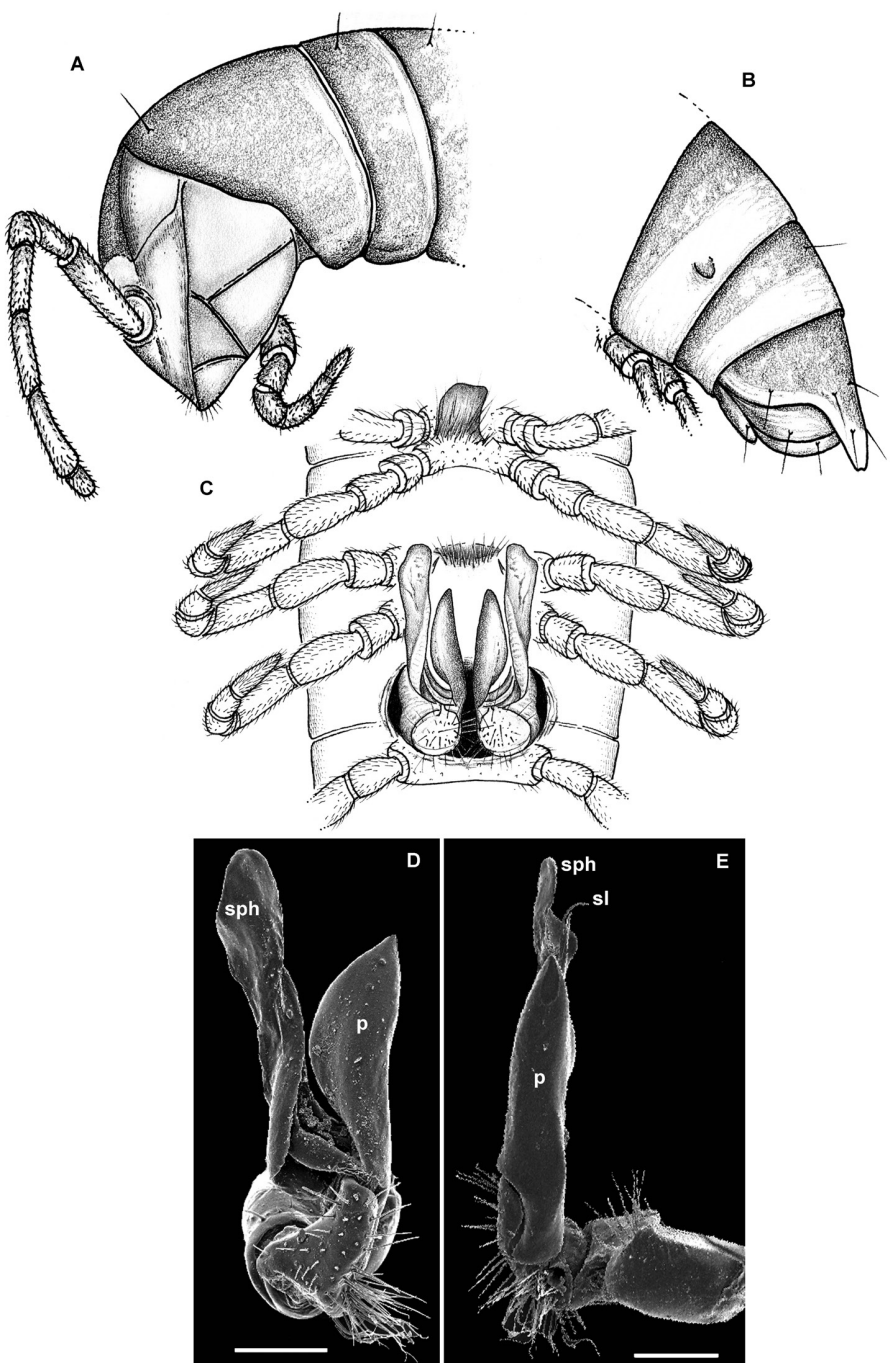
*Eviulisoma ngaia* sp. n., ♂ paratype. **A** anterior part of body, lateral view **B** posterior part of body, lateral view **C** body segments 5–7, ventral view **D, E** right gonopod, ventral and mesal views, respectively. Scale bars: 0.2 mm (**D, E**); **A–C**, drawn not to scale. Designations in text.

Differs from all congeners but *Eviulisoma ngaiaorum* sp. n. in the absence of a sternal excavation in ♂ segment 6, from *Eviulisoma ngaiaorum* sp. n. in the absence of sternal cones in the ♂ and by the presence of a well-developed, phylloid, postfemoral process of the gonopod (Fig. 1C–E). See also Key below.

#### Description.

Length of holotype ca 16 (♂), of adult paratype ca 18 mm (♀), width of midbody metazonae 1.5–1.6 (♂) or 2.0 mm (♀). Coloration uniformly yellowish, often with an annulated pattern of slightly more intense yellowish to marbled reddish yellow metazonae. Legs usually slightly lighter to nearly pallid.

Body subcylindrical, metazonae only faintly vaulted laterally compared to prozonae (Fig. 1A, B). In width, collum > segment 2 > head = segments 5-16 > 3 = 4 (♂) or head = segments 6-16 > 2 = 4 (♀); body behind segment 17 gradually tapering towards telson. Clypeolabral region rather densely setose, vertigial region bare (Fig. 1A). Antennae medium-sized, only slightly clavate, reaching behind body segment 2 (♂) or its midpoint (♀) when stretched dorsally; in length, antennomere 2 = 3 = 6 > 4 = 5 > 1 = 7; antennomeres 5 and 6 each with a distodorsal compact group of tiny bacilliform sensilla (as in Fig. 4G). Paraterga nearly missing, on each side a large, broadly rounded, ventrolateral lobe only in collum; a modest, caudally invariably rounded ridge demarcated by a premarginal lateral sulcus only dorsally in segment 2, thereafter totally wanting (Fig. 1A, B). Ozopores lateral, rather inconspicuous (as in Fig. 4B, C), lying at ca 1/3 of metazonite length in front of caudal margin (Fig. 1B). Body surface dull to poorly shining, smooth, microalveolate to faintly shagreened. Axial line missing. A transverse metatergal pigmented line traceable only dorsally in caudal 1/3 on segments 5–18, absent from 19^th^. Tergal setae short, mostly ca 1/4–1/5 as long as metazonite, largely abraded, pattern traceable only as 2+2 or 3+3 setae, but not their insertion points, placed in anterior 1/3 of metaterga. Stricture dividing pro- and metazonae rather thin, shallow, smooth. Pleurosternal carinae rather evident, arcuate ridges devoid of a caudal tooth, visible until segment 10 (♂) or 7 (♀). Epiproct (Fig. 1B) long, flattened dorsoventrally, very faintly concave apically, subapical lateral papillae small, but evident, removed unusually far forward from tip. Hypoproct nearly semi-circular, caudal 1+1 setae clearly separated, borne on minute knobs and clearly removed from caudal margin.

Sternites generally without modifications, densely setose, cross-impressions evident, but axial impressions especially weak; a subquadrate, densely setose lobe between ♂ coxae 4 (Fig. 1C), sternite between ♂ coxae 5 caudally, sterna between ♂ coxae 6 and 7 entirely and clearly flattened. Legs densely setose, rather short, with neither adenostyles nor dorsally bulged prefemora, 1.1–1.2 (♂) or 0.9–1.0 (♀) times as long as body height; ♂ tibial and tarsal brushes consisting of modified setae (as in Fig. 4D–F), present until a few last leg-pairs, tibiae thereby being a little, but clearly shorter than tarsi; ♀ tarsi ca 1.5 times as long as tibiae (as in Fig. 4B).

Gonopods (Fig. 1C–E) compact, with a lamellar solenophore (**sph**) (= tibiotarsus in Jeekel’s (2003) terminology) about as long as a flagelliform solenomere (**sl**), both being considerably higher than a simple, phylloid, postfemoral process (**p**).

Vulvae densely setose, without peculiarities, as in Fig. 4M, N.

#### Remarks.

Due to flattened, not deeply excavate, sterna between ♂ coxae 6 and 7, this species resembles *Eoseviulisoma* Brolemann, 1920, but the presence of a central lobe between ♂ coxae 4 warrants the assignment of this species to *Eviulisoma*. Brolemann (1920: 163) diagnosed *Eoseviulisoma* as follows.

«Sous-genre *Eviulisoma*, s. str. — Un prolongement entre les pattes de la 4e paire. Une excavation sternale accentuée au 6e segment. — Tronc du télopodite des gonopodes plus court que les rameaux. Suture transverse des métazonites lisse. — Type: *E. Cavalli* Silv.

Sous-genre *Eoseviulisoma*, nov. — Pas de prolongement entre les pattes de la 4e paire. — Excavation sternale du 6e segment très faible. — Tronc du télopodite des gonopodes plus long que les rameaux. — Suture transverse des métazonites perlée. — Type: *Eviulisoma julinum* Att.»

**Map 1.**
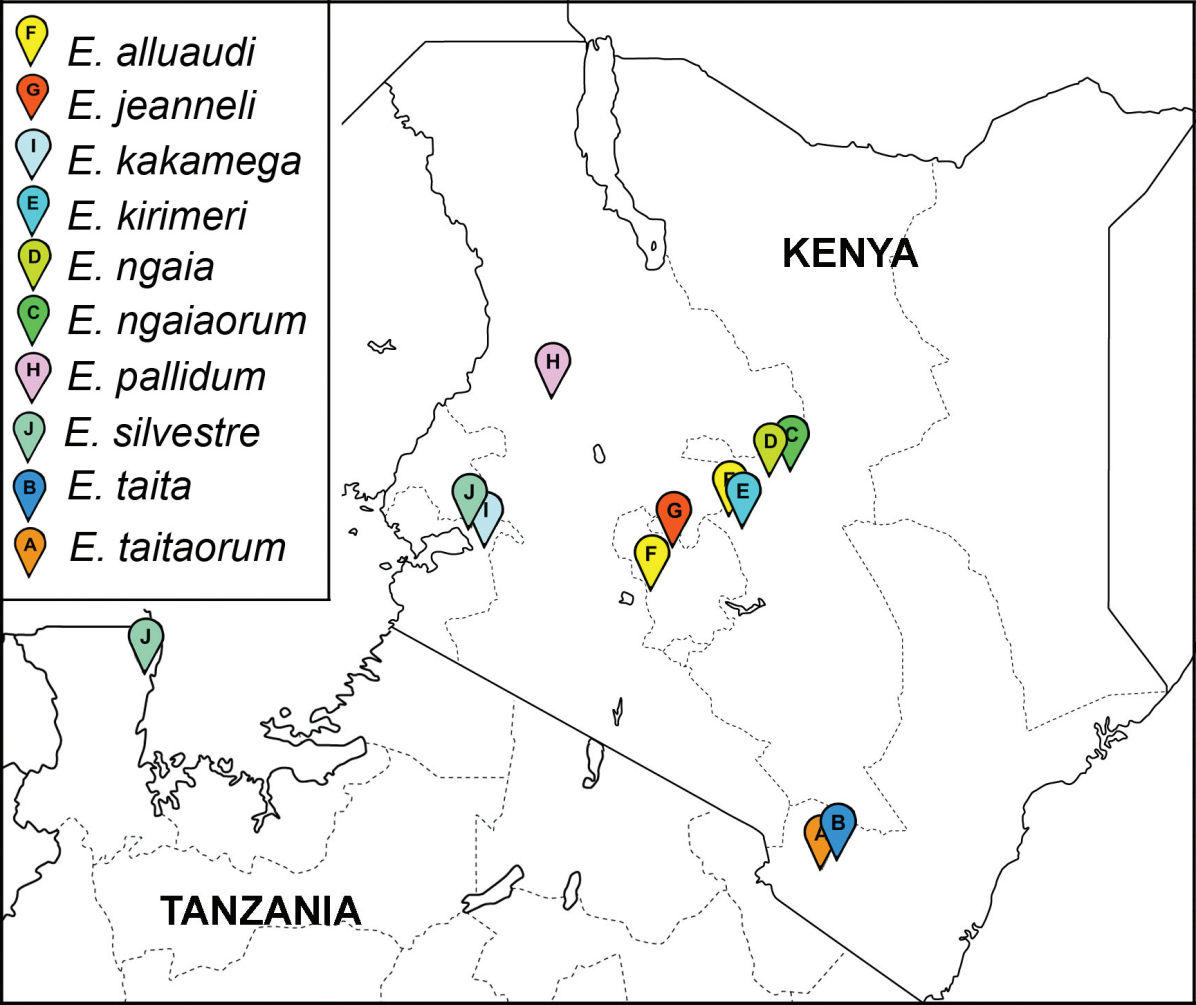
Distribution of *Eviulisoma* species in Kenya: *Eviulisoma taitaorum* sp. n. (**A**), *Eviulisoma taita* sp. n. (**B**), *Eviulisoma ngaiaorum* sp. n. (**C**), *Eviulisoma ngaia* sp. n. (**D**), *Eviulisoma kirimeri* sp. n. (**E**), *Eviulisoma alluaudi* Brolemann, 1920 (**F**), *Eviulisoma jeanneli* Brolemann, 1920 (**G**), *Eviulisoma pallidum* Attems, 1939 (**H**), *Eviulisoma kakamega* sp. n. (**I**), *Eviulisoma silvestre* (Carl, 1909) (**J**).

### Eviulisoma ngaiaorum sp. n.

Taxon classificationAnimaliaPolydesmidaParadoxosomatidae

http://zoobank.org/6119F18D-FF35-4C1C-A46E-35E1F8FD51B0

Fig. 2 , Map 1

#### Type material.

Holotype ♂ (MRAC 20806), Kenya, Ngaia Forest, N00°19', E38°02', ca 1070 m a.s.l., 3.XII.2002, leg. D. VandenSpiegel.

Paratypes: 1 ♂ fragment, 1 ♂ subadult, 8 juv. (MRAC 20806), same data, together with holotype.

#### Name.

To emphasize the type locality, in Latin meaning “a dweller of Ngaia”.

#### Diagnosis.

**Figure 2.**
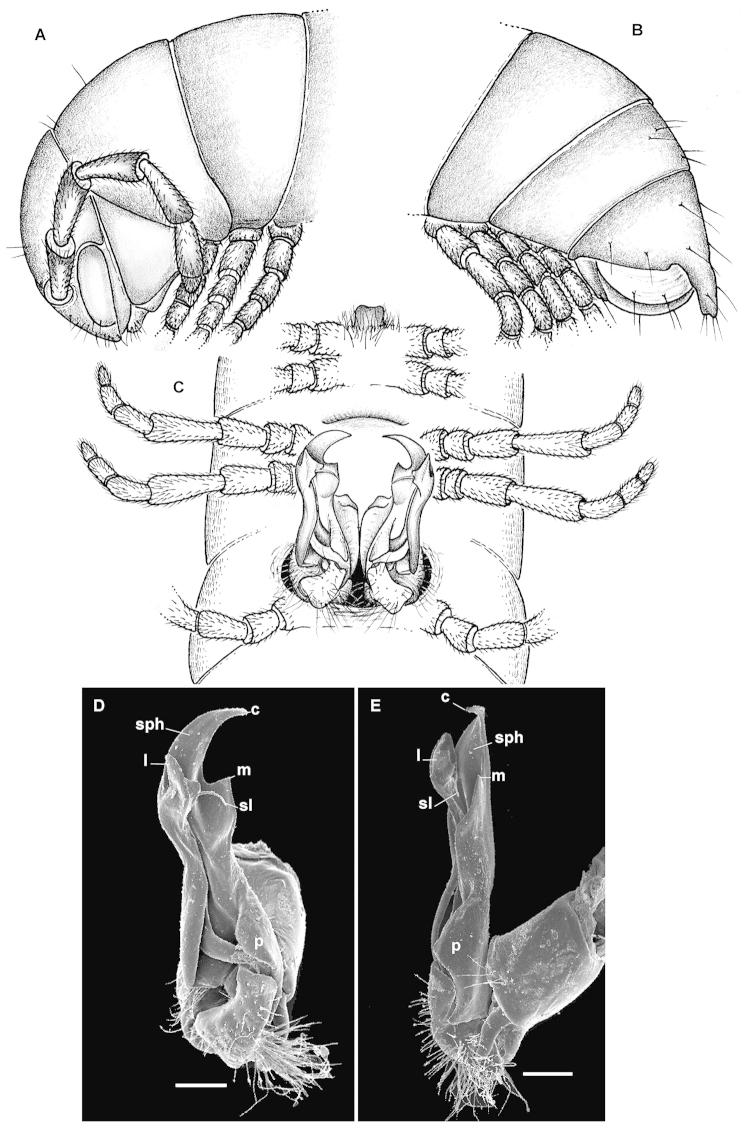
*Eviulisoma ngaiaorum* sp. n., ♂ holotype (**A–C**) & ♂ paratype (**D, E**). **A** anterior part of body, lateral view **B** posterior part of body, lateral view **C** body segments 5–7, ventral view **D, E** right gonopod, ventral and mesal views, respectively. Scale bars: 0.1 mm (**D, E**); **A–C**, drawn not to scale. Designations in text.

Differs from all congeners but *Eviulisoma ngaia* sp. n. in the absence of a sternal excavation in ♂ body segment 6, from *Eviulisoma ngaia* sp. n. in the presence of sternal cones in the ♂ and only a vestigial gonopod postfemoral process (Fig. 2C–E). See also Key below.

#### Description.

Length of adults ca 20 mm (♂ holotype), width of midbody metazonae 2.2 mm (both ♂ holotype and ♂ fragment paratype). Juveniles entirely pallid.

Coloration and other adult characters as in *Eviulisoma ngaia* sp. n., except as follows.

Transverse metatergal sulcus/line wanting. Tergal setae mostly retained, pattern 3+3 (Fig. 2B). Pleurosternal carinae rather evident, arcuate ridges devoid of a caudal tooth, visible until segment 15 (♂). Epiproct (Fig. 2B) subtruncate apically, subapical lateral papillae rather large and only poorly removed from tip.

Sternites behind gonopods with a distinct sharp cone near each ♂ coxa, each caudal pair per diplosegment being a little stronger than anterior one. Setose lobe between ♂ coxae 4 (Fig. 2C) faintly concave at tip. Sterna between ♂ coxae 6 and 7 (Fig. 2C) clearly flattened, their coxae being a little enlarged and conical distoventrally. Legs densely setose, rather short, 1.2–1.3 times as long as body height (♂), tibiae behind gonopods thereby being mostly subequal in length to tarsi; ♂ tibiae and tarsi with ventral brushes until last two leg-pairs.

Gonopods (Fig. 2C–E) with a lamellar, lateroparabasally strongly expanded solenophore (**sph**) carrying a large apical claw and two pre-apical teeth, one mesal (**m**), the other lateral (**l**); a flagelliform solenomere (**sl**) about as long as to reach bases of both **l** and **m**; postfemoral process (**p**) very short, fold-shaped, vestigial.

### Eviulisoma taitaorum sp. n.

Taxon classificationAnimaliaPolydesmidaParadoxosomatidae

http://zoobank.org/83C83C70-46C1-4AC7-9D13-EEA08A149E11

**Figure 3.**
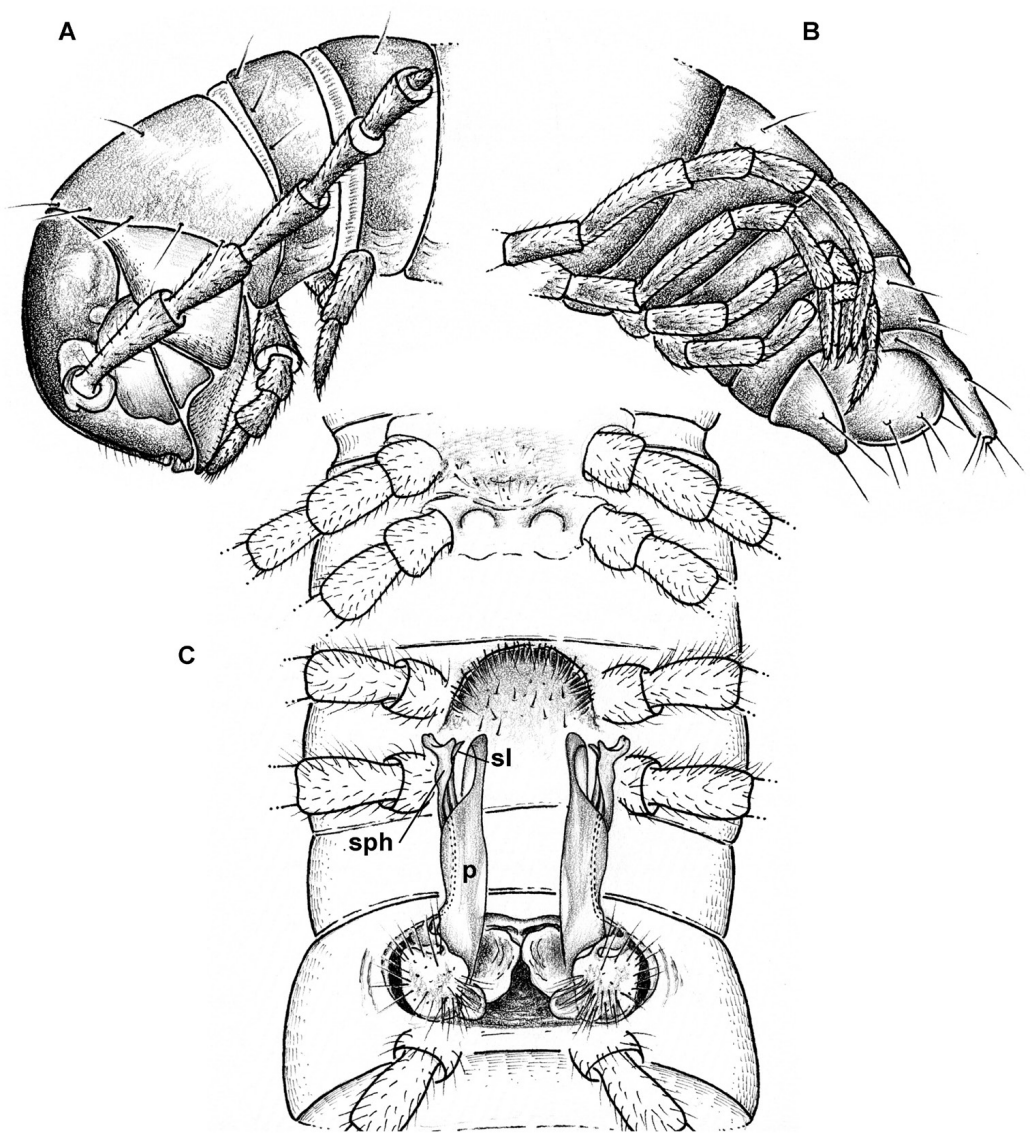
*Eviulisoma taitaorum* sp. n., ♂ paratype. **A** anterior part of body, lateral view **B** posterior part of body, lateral view **C** body segments 5–7, ventral view. Drawn not to scale. Designations in text.

**Figure 4.**
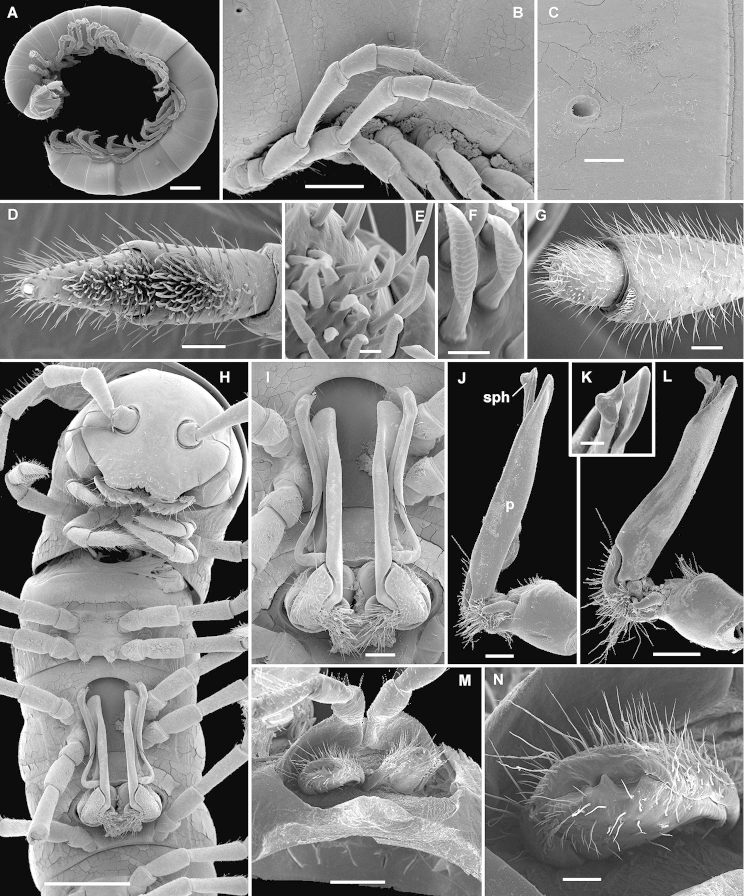
*Eviulisoma taitaorum* sp. n., ♂ (**A, D–L**) & ♀ paratypes (**B, C, M, N**). **A** habitus, lateral view **B** midbody legs, lateral view **C** ozopore, lateral view **D** ventral brushes on tibia and tarsus, ventral view **E, F** modified setae of ventral brushes, ventral view **G** antennomeres 6–8, sublateral view **H** anterior part of body, ventral view **I** both gonopods in situ, ventral view **J, L** right gonopod, mesal and submesal views, respectively **K** gonopod tip, sublateral view **M** both vulvae in situ, ventrocaudal view **N** right vulva, ventrocaudal view. Scale bars: 1.0 (**A**), 0.5 (**B, H, M**), 0.2 (**I, J, L**), 0.1 (**C, D, G, K, N**) & 0.01 mm (**E, F**). Designations in text.

**Map 2.**
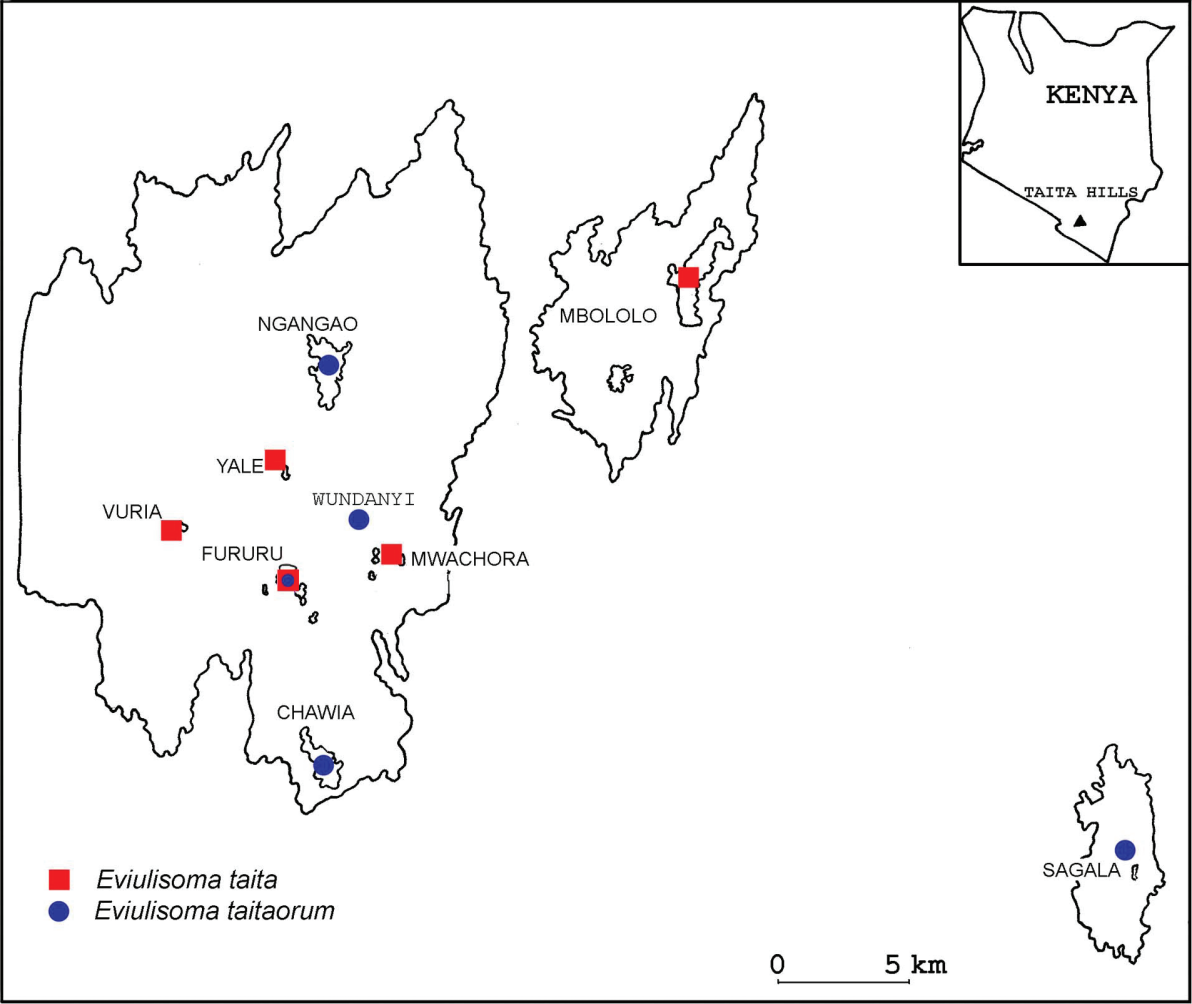
Distribution of *Eviulisoma taitaorum* sp. n. (blue dot) and *Eviulisoma taita* sp. n. (red square) in the Taita Hills, Kenya.

Figs 3 , 4 , Maps 1 , 2

#### Type material.

Holotype ♂ (MRAC 22630), Kenya, Taita Hills, Chawia Forest, 1500 m a.s.l., S03°29', E38°20', pitfall trapping, 1–20.VI.1999, leg. R. Mwakos.

Paratypes: 3 ♂, 1 ♀, 8 juv. (MRAC 18071), same data, together with holotype; 3 ♂ (MRAC 18016), same locality, 1500 m a.s.l., pitfall traps, 10–26.VI.1999, leg. R. Mwakos; 3 ♀ (MRAC 18096), same locality, 1500 m a.s.l., pitfall traps, III–IV.1999, leg. L. Rogo; 1 ♀, 3 juv. (MRAC 17993), same locality, 1500 m a.s.l., III–IV.1999, leg. D. VandenSpiegel; 3 juv. (MRAC 18505), same locality, Winkler extraction, 7.XII.1999, leg. D. VandenSpiegel & J. P. Michiels; 1 ♂, 2 ♀, 5 juv. (MRAC 18424), same locality, 7.XII.1999, leg. D. VandenSpiegel & J. P. Michiels; 1 ♂ fragment, 1 ♀, 2 juv. (MRAC 18043), 1 ♂, 1 ♀ (ZMUM ρ2443), Taita Hills, Ngangao Forest, S03°22', E38°21', 1820 m a.s.l., 17.VIII.1999, leg. R. Mwakos; 1 ♂ fragment, 20 ♀ (MRAC 18476), same locality, 4.XII.1999, leg. D. VandenSpiegel & J. P. Michiels; 3 ♀, 3 juv. (MRAC 18008), same locality, 1820 m a.s.l., 19.VI.1999, leg. D. VandenSpiegel; 1 ♀ (MRAC 18090), same locality, 1820 m a.s.l., pitfall traps, III–IV.1999, leg. D. VandenSpiegel, 1 ♂ (MRAC 18036), same locality, 1820 m a.s.l., pitfall traps, 15–17.III.1999, leg. L. Rogo; 1 ♂ (MRAC 22622), Taita Hills, Fururu Forest, S3°26', E38°20', 9.XII.1999, leg. D. VandenSpiegel & J. P. Michiels; 1 ♂, 1 ♀, 7 juv. (MRAC 22623), same locality, pitfall traps, 14–17.XII.2004, leg. A. Bwong, J. Mwandoe & J. Measey; 1 ♂, 1 ♀, 3 juv. (MRAC 18083), Taita Hills, Vuria Forest, S03°24', E38°17', 2200 m a.s.l., 26.VI.1999, leg. D. VandenSpiegel; 1 ♀ (MRAC 18459), Taita Hills, Sagala Forest, S03°50', E38°58', 5.XII. 1999, leg. D. VandenSpiegel & J. P. Michiels.

Non-types: ca 30 juv. (MRAC 18.543), Taita Hills, Fururu Forest, S03°26', E38°20', Winkler extraction, 9.XII.1999; 1 ♀ (MRAC 18441), Taita Hills, Wundanyi, near house, S03°24'07", E38°21'49", 6.XII. 1999, all leg. D. VandenSpiegel & J. P. Michiels.

#### Name.

To emphasize the type locality, in Latin meaning “a dweller of Taita”.

#### Diagnosis.

Differs from all congeners in the remarkable size dimorphism, coupled with absence of a sternal lobe between ♂ coxae 4, as well as the subequally long and slender solenophore (**sph**) and postfemoral process (**p**) (Figs 3C, 4H–L). See also Key below.

#### Description.

Length of adults ca 17–20 (♂ holotype and some ♂ & ♀ paratypes from Chawia, Fururu and from Ngangao) or 28–38 mm (most of ♂ & ♀ paratypes from Fururu and Ngangao, all few paratypes from Sagala and Vuria), width of midbody metazonae 1.7–1.8 (♂ holotype and some ♂ paratypes) up to 2.0 mm (♀ paratypes from Chawia) or 2.5–2.6 (most of ♂ paratypes from Fururu and Ngangao) up to 3.0–3.8 mm (most of ♀ paratypes from Fururu and Ngangao).

Coloration from pallid, via light pinkish or marbled pinkish brown to nearly chocolate brown, pattern often annulated due to darker metazonae, including later instars of larger morph. Legs pallid to yellowish, earlier instars always entirely pallid. Sometimes a narrow, darker, pigmented axial line and a similar transverse line in caudal 1/3 of metaterga.

All characters as in *Eviulisoma ngaia* sp. n. (Fig. 4A–G, M, N), except as follows.

Surface rather smooth and shining (Figs 3, 4A–C, H), near ozopores faintly rugulose longitudinally, slightly microgranulate below in ♂. Hypoproct more narrowly rounded up to nearly pointed between 1+1 caudal setae (♂). Pleurosternal carinae faint (Fig. 4H), mostly line-shaped, visible until segment 17 (♂) or 15 (♀). Sterna between ♂ coxae 4 and 5 each with a pair of paramedian cones caudally, devoid of any central lobes (Figs 3C, 4H); sterna between ♂ coxae 6 and 7 unusually deeply excavate and ledge-shaped for accommodation of gonopod tips (Fig. 4H), the excavation’s frontal edge being densely setose (Fig. 3C). Postgonopodial sterna with small, but evident, sometimes pointed cones near each coxa, anterior pair being always smaller than caudal one on each diplosegment. ♂ tarsi either a little longer than tibiae (usually smaller morph) or both subequal in length (usually larger morph). Legs 1.2–1.4 (♂) or 0.8–0.9 (♀) times as long as body height. ♂ tibiae and tarsi with ventral brushes until last two leg-pairs, their setae being flattened, same as in other new species (Fig. 4D–F).

Gonopods (Figs 3C, 4H–L) very slender, with solenophore (**sph**), postfemoral process (**p**) and solenomere (**sl**) subequal in length.

Vulvae without peculiarities, as in Fig. 4M, N.

#### Remarks.

This new species seems remarkable in being represented by two different size morphs which invariably co-occur at least in sufficiently rich samples and show no intermediates. Thus, in one sample from Chawia the adult ♂♂ can vary in size by 1.5–2.0 times. Larger animals tend to be darker than smaller ones.

Such a strong size morphism could be advantageous for the local populations in variably adverse ecological conditions, possibly allowing selection for different life strategies.

The above two species from Taita Hills show parapatry (Map 1), co-occurring only in Fururu Forest.

In addition, the absence of a central lobe between ♂ coxae 4 is rather characteristic of *Eoseviulisoma* Brolemann, 1920, but the smooth metazonital suture, the structure of the gonopods and the deeply excavate sterna between ♂ coxae 6 and 7 warrant the assignment of this species to *Eviulisoma* (cf. Brolemann 1920). This is just another example that these two genera may well prove to be synonymous. Both Brolemann (1920) and Attems (1937) had treated *Eoseviulisoma* as only a subgenus of *Eviulisoma*, but Hoffman (1953) elevated the former to the rank of a full genus which currently includes only 2–3 species from Tanzania and the Democratic Republic of the Congo.

### Eviulisoma taita sp. n.

Taxon classificationAnimaliaPolydesmidaParadoxosomatidae

http://zoobank.org/2D851CA6-F810-4A2D-BE59-EC7EC6FB294E

**Figure 5.**
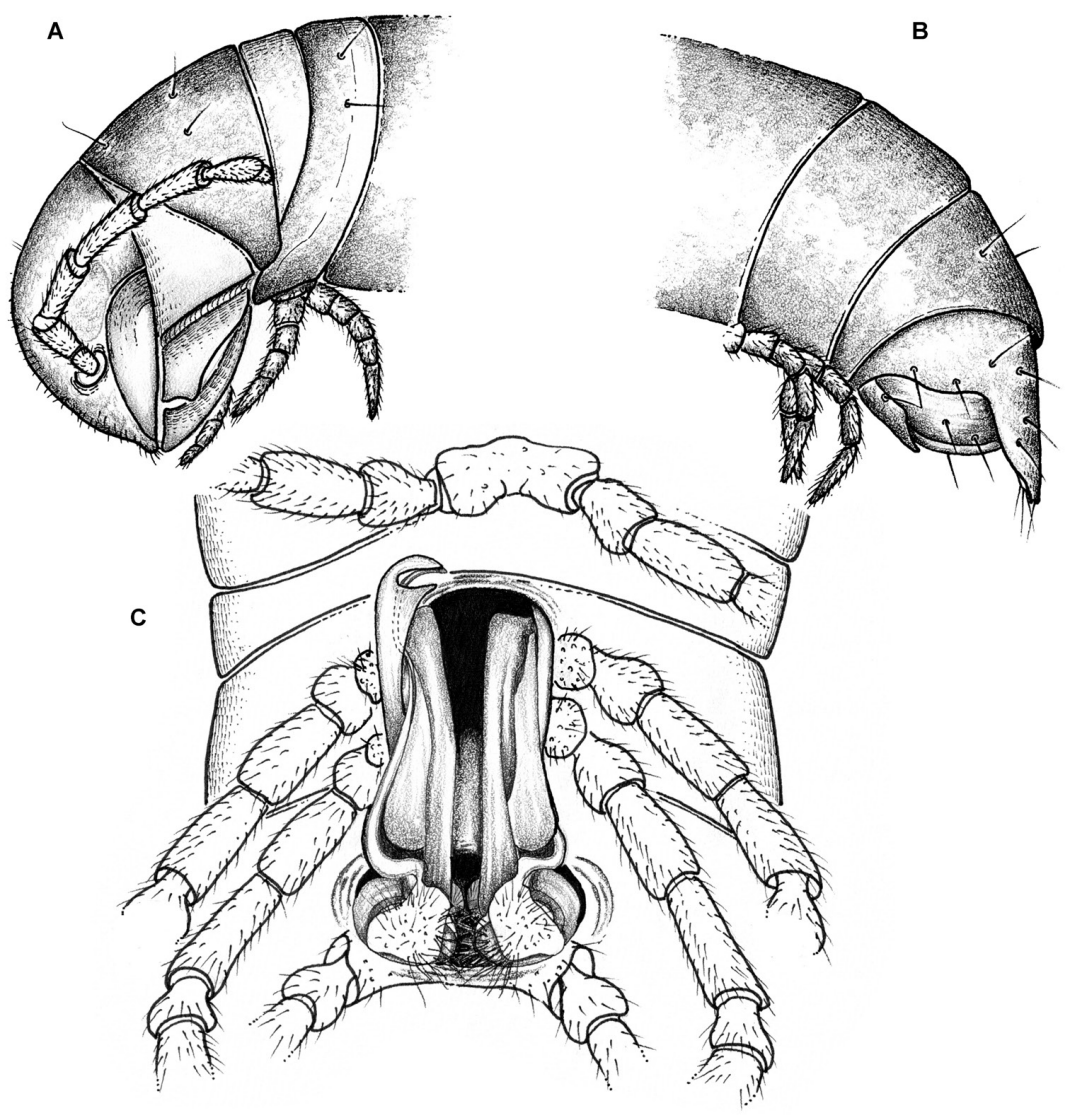
*Eviulisoma taita* sp. n., ♂ paratype. **A** anterior part of body, lateral view **B** posterior part of body, lateral view **C** body segments 5–7, ventral view. Drawn not to scale.

**Figure 6.**
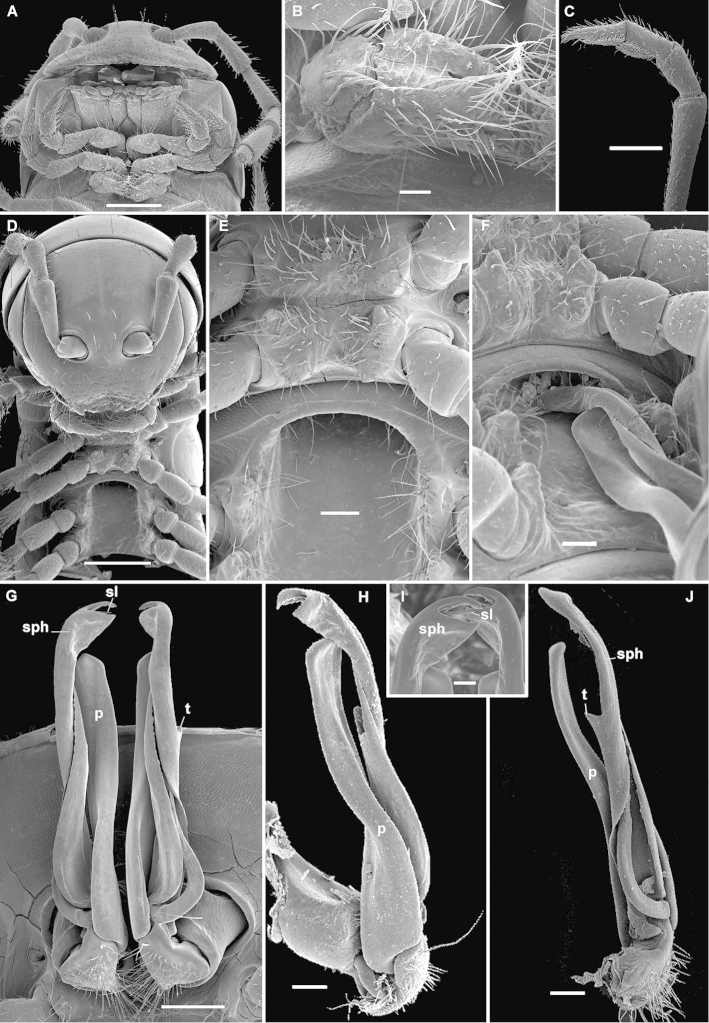
*Eviulisoma taita* sp. n., ♀ (**A, B**) & ♂ (**C–J**) paratypes. **A, D** anterior part of body, ventrocaudal and ventral views, respectively **B** right vulva, ventrocaudal view **C** distal part of a midbody leg, lateral view **E** sterna between coxae 4–7, ventral view **F** same, but with left gonopod placed into sternal pocket-shaped excavation **G** both gonopods in situ, ventral view **H, J** left gonopod, mesal and lateral views, respectively **I** tips of both gonopods in situ, ventral view. Scale bars: 0.5 (**A, C, D**), 0.2 (**G**), 0.1 (**E, F, H, J**) & 0.05 mm (**B, I**). Designations in text.

Figs 5 , 6 , Maps 1 , 2

#### Type material.

Holotype ♂ (MRAC 22631), Kenya, Taita Hills, Mbololo Forest, S03°22.56', E38°20.70', 1800–1900 m a.s.l., pitfall traps, III–IV.1999, leg. L. Rogo.

Paratypes: 17 ♂, 13 ♀, 4 juv. (MRAC 18084), 1 ♂, 1 ♀ (ZMUM ρ2444), same data, together with holotype; 2 ♂, 2 ♀ (MRAC 18029), same locality, pitfall traps, 3.VII-2.VIII.1999, leg. R. Mwakos; 1 ♂, 1 ♀ (MRAC 18412), same locality, 8.XII.1999, leg. D. VandenSpiegel & J. P. Michiels; 1 ♀, 1 juv. (MRAC 17990), same locality, 22.VI.1999, leg. D. VandenSpiegel; 9 ♂, 8 ♀, 33 juv. (MRAC 18039), same locality, 1800–1900 m a.s.l., sieving, 2–10.VII.1999, leg. R. Mwakos; 1 ♀ (MRAC 17976), same locality, 21.VI.1999, leg. D. VandenSpiegel; 1 ♂, 1 ♂ fragment, 1 ♀, 1 ♀ fragment (MRAC 18414), same locality, 8.XII.1999, leg. D. VandenSpiegel & J. P. Michiels; 3 ♂, 1 ♀ (MRAC 18100), Taita Hills, Yale Forest, 1840 m, S03°39', E38°33', pitfall traps, III–IV.1999, leg. L. Rogo; 4 ♂, 4 ♀, 22 juv. (MRAC 18451), Taita Hills, Fururu Forest, S03°26', E38°20', 9.XII.1999; 1 ♂, 3 ♀, 20 juv. (MRAC 18495), same locality, Winkler extraction, 9.12.1999; 5 ♂, 4 ♀, 1 juv. (MRAC 18576), Taita Hills, Mwachora Forest, Winkler extraction, 10.XII.1999, all leg. D. VandenSpiegel & J. P. Michiels; 2 ♂ (MRAC 22632), same data; 1 ♂, 1 ♀, 1 ♀ fragment, 1 juv. (MRAC 22633), same locality, 15.II.2004, leg. T. Spanhove & M. Chovu.

#### Name.

To emphasize the type locality, a noun in apposition.

#### Diagnosis.

Differs from congeners by a broadly and regularly rounded hypoproct, coupled with the presence of sternal cones behind ♂ body segment 7, and the lamellar, slender, apically unciform and bidentate solenophore (**sph**) carrying a lateral tooth midway (**t**) and reaching about as long as a flagelliform solenomere (**sl**), both **sph** and **sl** being considerably higher than a rather simple, similarly slender, postfemoral process (**p**). See also Key below.

#### Description.

Length of adults ca 16–23 (♂) or 18–28 mm (♀), width of midbody metazonae 1.5–2.7 (♂) or 2.0–3.7 mm (♀). Holotype ca 16 mm long and 1.6 mm wide on midbody metazonae.

Coloration from pallid to annulated chocolate brown due to darker metazonae, often with a thin axial pigment line and a similar transverse pigment line in posterior 1/3 of metaterga.

Other adult characters as in *Eviulisoma ngaia* sp. n., except as follows.

Vertigial region with a few setae (Figs 5A, 6D). Stricture between pro- and metazonae very delicately striolate. Tegument generally smooth, often with only a few arcuate striae near and below ozopores. Pleurosternal carinae rather evident, arcuate ridges devoid of a caudal tooth, visible until segment 16 (♂, ♀). Epiproct long (Fig. 5B), faintly concave apically, subapical lateral papillae evident, well removed from tip. Hypoproct broadly rounded.

Setose lobe between ♂ coxae 4 (Fig. 6E) low, subtrapeziform, slightly rounded apically. Sternite between ♂ coxae 5 densely setose, with paramedian cones caudally (Fig. 6E); sterna between ♂ coxae 6 and 7 unusually deeply excavate and ledge-shaped for accommodation of gonopod tips (Figs 5C, 6D–F), the excavation’s frontal edge being sparsely setose (Fig. 6E). Postgonopodial sterna mostly with small, low, blunt cones near each coxa, anterior pair being even smaller than caudal one on each diplosegment. ♂ tarsi considerably to only slightly longer than tibiae (Fig. 6C). Legs 1.5–1.6 (♂) or 0.9–1.1 (♀) times as long as body height. ♂ tibiae and tarsi with ventral brushes until last two leg-pairs (Fig. 6C).

Gonopods (Figs 5C, 6F–J) with a lamellar, slender, apically unciform and bidentate solenophore (**sph**) carrying a lateral tooth midway (**t**) and being about as long as a flagelliform solenomere (**sl**), both **sph** and **sl** considerably higher than a rather simple, similarly slender, postfemoral process (**p**).

Vulvae without peculiarities, as in Fig. 6A, B.

### Eviulisoma kirimeri sp. n.

Taxon classificationAnimaliaPolydesmidaParadoxosomatidae

http://zoobank.org/D7ED4341-A7DE-494E-9041-21EF9E026D42

Fig. 7 , Map 1

#### Type material.

Holotype ♂ (MRAC 22624), Kenya, Kirimeri Forest near Runyenyere, S00°25', E37°33', 1700 m a.s.l., sieved litter, 27.IV.2004, leg. D. VandenSpiegel, R. Jocqué & C. Warui.

Paratype: 1 ♂ (MRAC 22625), same data, together with holotype.

#### Name.

To emphasize the type locality, a noun in apposition.

#### Diagnosis.

**Figure 7.**
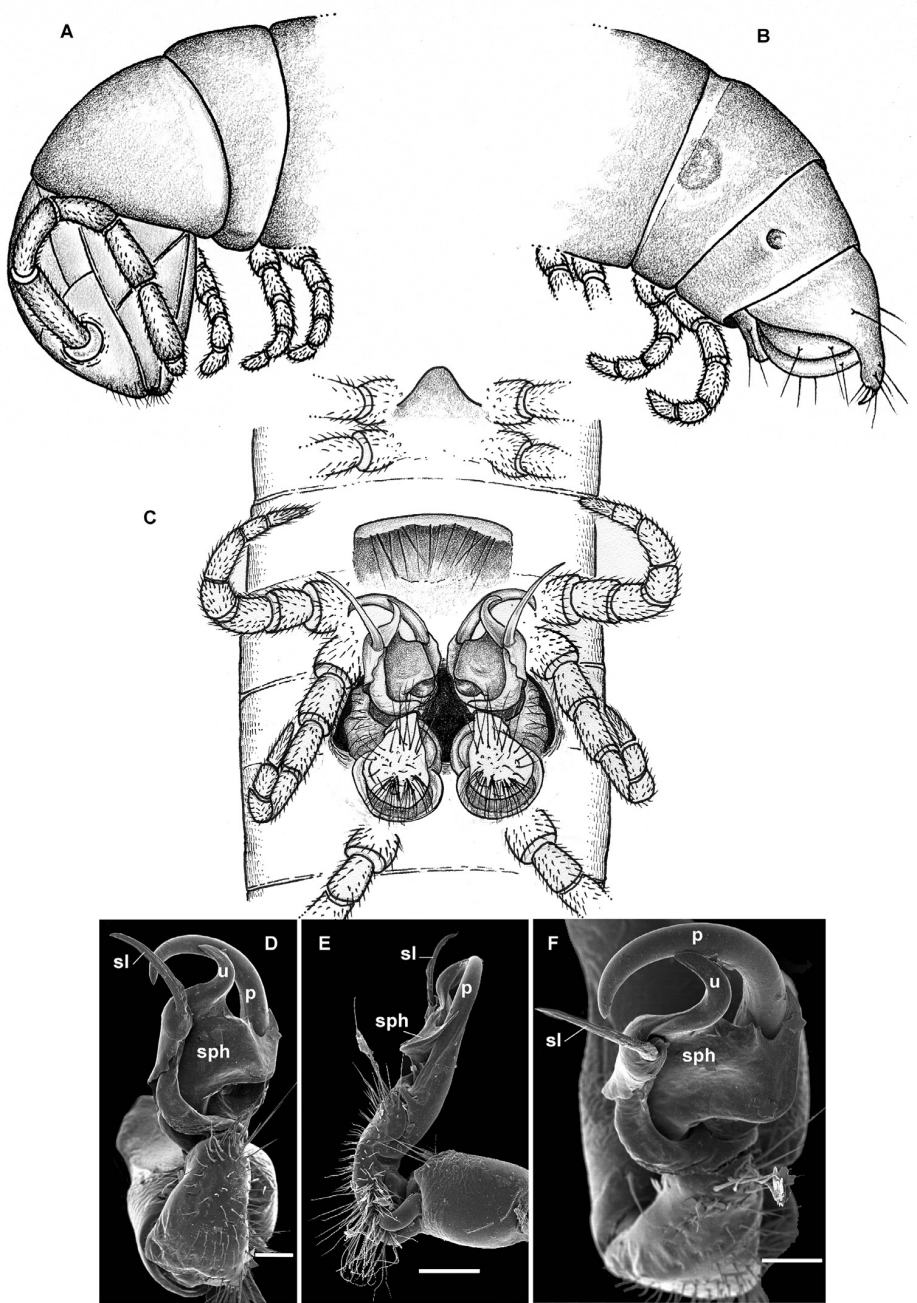
*Eviulisoma kirimeri* sp. n., ♂ paratype. **A** anterior part of body, lateral view **B** posterior part of body, lateral view **C** body segments 5–7, ventral view **D–F** left (**D, F**) and right (**E**) gonopod, ventral, mesal and anteroventral views, respectively. Scale bars: 0.2 (**E**) & 0.1 mm (**D, F**); **A–C**, drawn not to scale. Designations in text.

Differs from congeners in the epiproct showing two distinct apical claws directed ventrad (Fig. 7b), as well as the gonopods being divergent, rather loose, with a complex, lamellar, apically unciform (**u**) solenophore (**sph**) partly sheathing a longer flagelliform solenomere (**sl**); postfemoral process (**p**) very simple, sickle-shaped (Fig. 7C–F). See also Key below.

#### Description.

Length of ca 15–16 mm, width of midbody metazonae 1.5 (♂ holotype) or 1.7 mm (♂ paratype). Coloration entirely pallid.

Other adult characters as in *Eviulisoma ngaia* sp. n., except as follows.

Clypeolabral region rather sparsely setose (Fig. 7A). Stricture between pro- and metazonae very delicately striolate. Tegument generally smooth, often with only a few arcuate striae near and below ozopores. Pleurosternal carinae rather evident, arcuate ridges devoid of a caudal tooth, visible until segment 15 (♂). Epiproct (Fig. 7B) faintly concave between two evident, claw-shaped, apical papillae directed ventrad; subapical lateral papillae evident, rather well removed from tip. Hypoproct subtriangular, pointed between 1+1 submarginal setae borne on minute knobs.

Setose lobe between ♂ coxae 4 (Fig. 7C) roundly subtriangular. Sternite between ♂ coxae 5 flattened; sterna between ♂ coxae 6 and 7 unusually deeply excavate and ledge-shaped for accommodation of gonopod tips, the excavation’s frontal edge being densely setose (Fig. 7C). Postgonopodial sterna with small, but evident, almost sharp cones near each coxa, anterior pair being smaller than caudal one on each diplosegment. ♂ tarsi largely considerably longer than tibiae (Fig. 7C). Legs 1.2–1.3 times as long as body height (♂). All ♂ telopodite segments distal to coxa or prefemur with dense ventral brushes, but last leg-pair with ventral brushes retained only on tibiae and tarsi.

Gonopods (Fig. 7C–F) rather loose, divergent, with a complex, lamellar, apically unciform (**u**) solenophore (**sph**) partly sheathing a longer and flagelliform solenomere (**sl**); postfemoral process (**p**) very simple, strong and sickle-shaped.

### Eviulisoma kakamega sp. n.

Taxon classificationAnimaliaPolydesmidaParadoxosomatidae

http://zoobank.org/C175D502-7342-4456-9B79-C73CD155A752

Figs 8 , 9 , Map 1

#### Type material.

Holotype ♂ (incomplete, only head and first 13 segments present) (MRAC 20771), Kenya, Likhanda Hills, Kakamega Forest, S00°13', E34°54', pitfall traps, 5.II.2002, leg. D. S. Smith.

Paratypes: 1 ♂ (incomplete, lacking gonopods and five posteriormost segments), 4 ♀, 5 juv., 1 fragment (MRAC 20772), same data, together with holotype.

#### Name.

To emphasize the type locality, a noun in apposition.

#### Diagnosis.

**Figure 8.**
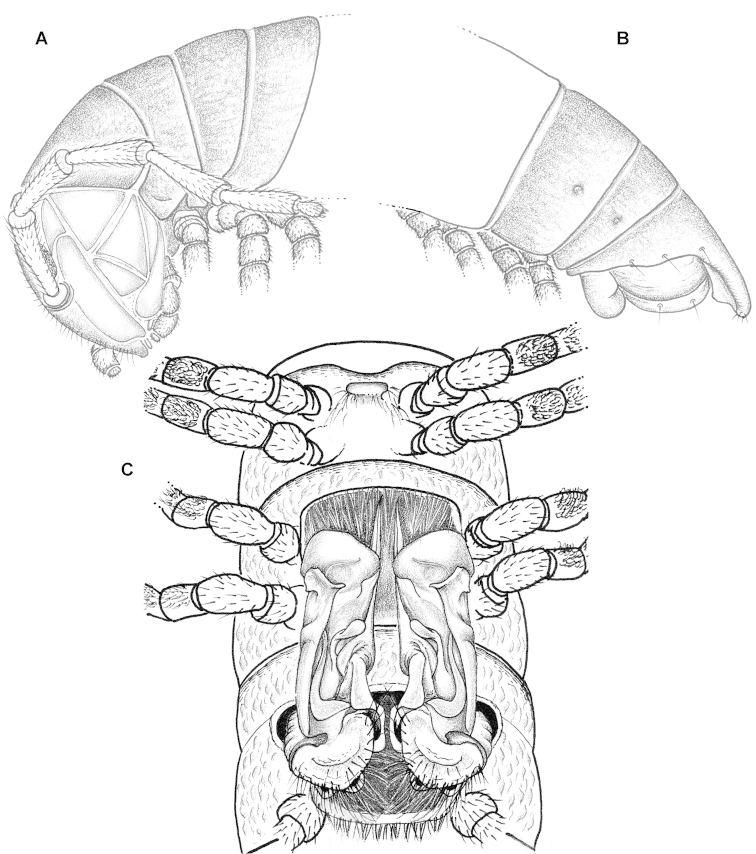
*Eviulisoma kakamega* sp. n., ♂ holotype (**A, C**) & ♀ paratype (**B**). **A** anterior part of body, lateral view **B** posterior part of body, lateral view **C** body segments 5–7, ventral view. Drawn not to scale.

**Figure 9.**
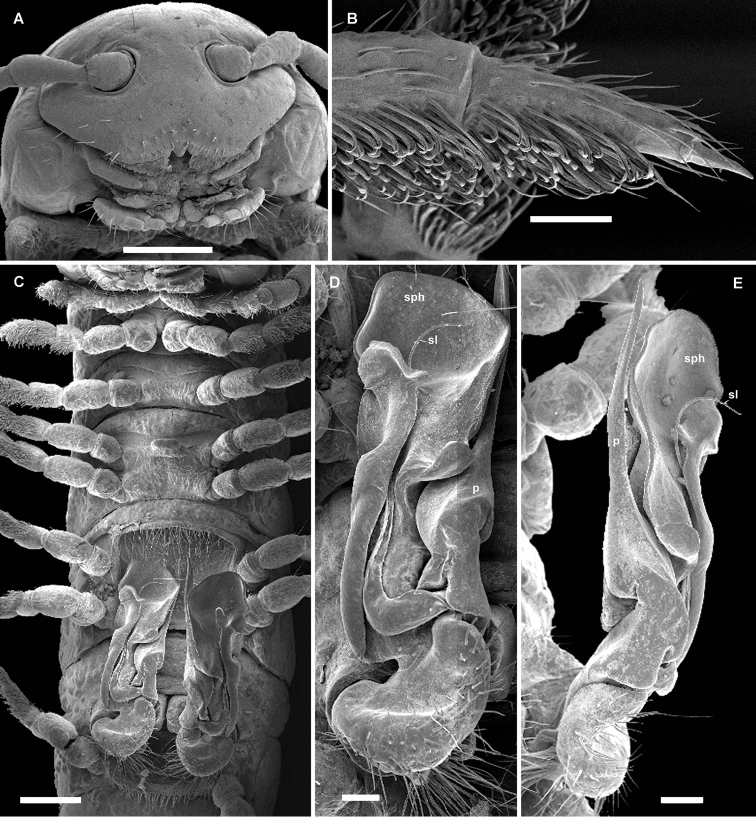
*Eviulisoma kakamega* sp. n., ♂ paratype. **A** anterior part of body, ventral view **B** ventral brushes on tibia and tarsus, lateral view **C** body segments 2–7, ventral view **D, E** right gonopod, ventral and lateral views, respectively. Scale bars: 0.5 (**A, C**), 0.2 (**D, E**) & 0.1 mm (**B**). Designations in text.

Differs from congeners by the gonopod solenophore (**sph**) being complex, cup-shaped, lamellar, about as long as a flagelliform solenomere (**sl**), flanked medially by a long, subspiniform, postfemoral process (**p**) (Figs 8C, 9C–E). See also Key below.

#### Description.

Length of ♀ ca 22–23 mm, width of midbody metazonae 2.1 (♂ holotype), 2.7 (♂ paratype) or 3.1–3.3 mm. Coloration uniformly light pinkish yellow, legs lighter yellow.

Other adult characters as in *Eviulisoma ngaia* sp. n., except as follows.

Vertigial region with a few setae (Figs 8A, 9A). Stricture between pro- and metazonae very delicately striolate. Tegument generally smooth, often with only a few arcuate striae near and below ozopores. Pleurosternal carinae rather evident, arcuate ridges devoid of a caudal tooth, visible at least until segment 15 (♂, ♀). Epiproct long (Fig. 8B), faintly concave between two small apical papillae, subapical lateral papillae evident, only slightly removed from tip (♀). Hypoproct semi-circular, regularly and broadly rounded, 1+1 submarginal setae borne on minute knobs and a little removed from margin.

Setose lobe between ♂ coxae 4 (Figs 8C, 9C) low, broad, clearly concave apically. Sternite between ♂ coxae 5 slightly elevated due to small caudolateral cones (Fig. 9C); sterna between ♂ coxae 6 and 7 unusually deeply excavate and ledge-shaped for accommodation of gonopod tips, the excavation’s frontal edge being densely setose (Figs 8C, 9C). Postgonopodial sterna with small, but evident, often sharp cones near each coxa, anterior pair being smaller than caudal one on each diplosegment. ♂ tarsi considerably longer than tibiae. Legs 1.4–1.5 (♂) or 1.1–1.2 (♀) times as long as body height. Dense ventral brushes on ♂ tibiae and tarsi present (Fig. 9B).

Gonopods (Figs 8C, 9C–E) rather compact, highly complex due to an apically cup-shaped, lamellar solenophore (**sph**) about as long as a flagelliform solenomere (**sl**), flanked medially by a long, subspiniform postfemoral process (**p**).

### Eviulisoma alluaudi

Taxon classificationAnimaliaPolydesmidaParadoxosomatidae

Brolemann, 1920

Fig. 10 , Map 1

#### Material.

3 ♂, 16 ♀, 4 juv. (MRAC 22626), 1 ♂, 1 ♀ (ZMUM ρ2445), Kenya, Chogoria Forest, S0°11'13", E37°28'07", 2658 m a.s.l., bamboo forest, sieved litter and beaten from bamboos, 24.IV.2004, leg. D. VandenSpiegel, R. Jocqué & C. Warui.

#### Remarks.

**Figure 10.**
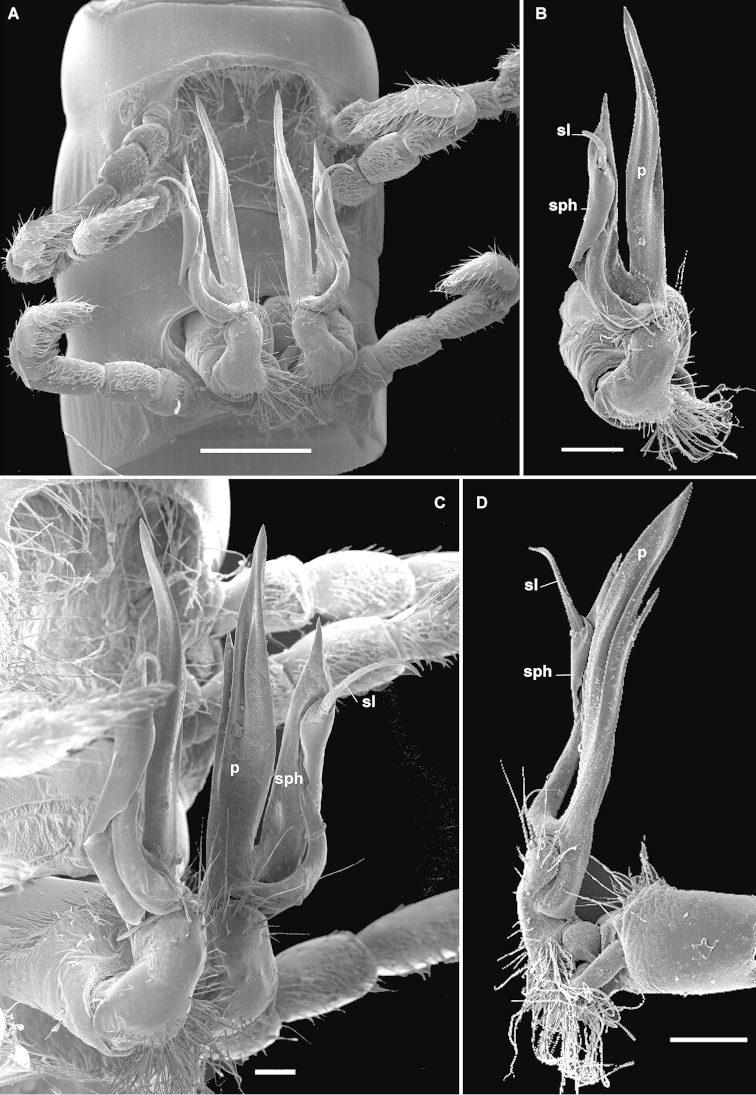
*Eviulisoma alluaudi* Brolemann, 1920, ♂ from Chogoria Forest. **A, C** body segments 6 & 7, ventral and ventrolateral views, respectively **B, D** right gonopod, ventral and mesal views, respectively. Scale bars: 0.5 (**A, C**), 0.2 (**D, E**) & 0.1 mm (**B**). Designations in text.

The above is only the second record of this species beyond the type locality: alpine meadows and a forest at 3100 m and 2600 m a.s.l., respectively, on Mt. Kinangop, S00°11', E37°28', Aberdare Ridge, Kenya (Brolemann 1920, Attems 1939). Even though the new samples, which are in rather poor condition, fully match Brolemann’s (1920) excellent original description, we provide additional illustrations (Fig. 10) to document the identity of this obviously high-montane species which appears to be more widely distributed at least in Kenya (Map 1). The shapes and proportions of the solenophore (**sph**), solenomere (**sl**) and postfemoral process (**p**) are quite characteristic.

### Eviulisoma silvestre

Taxon classificationAnimaliaPolydesmidaParadoxosomatidae

(Carl, 1909)

Fig. 11 , Map 1

#### Material.

1 ♂, 1 ♂ (incomplete, only last 8 segments present) (MRAC 22627), Kenya, Likhanda Hills, Kakamega Forest, S00°13', E34°54', pitfall traps, 28.IX.2002; 1 ♂ (incomplete, only segments 8–20 present) (MRAC 22628), same locality, pitfall traps, 6.IV.2002; 1 ♂ (MRAC 22629), same locality, pitfall traps, 6.VII.2002, all leg. D. S. Smith.

#### Remarks.

**Figure 11.**
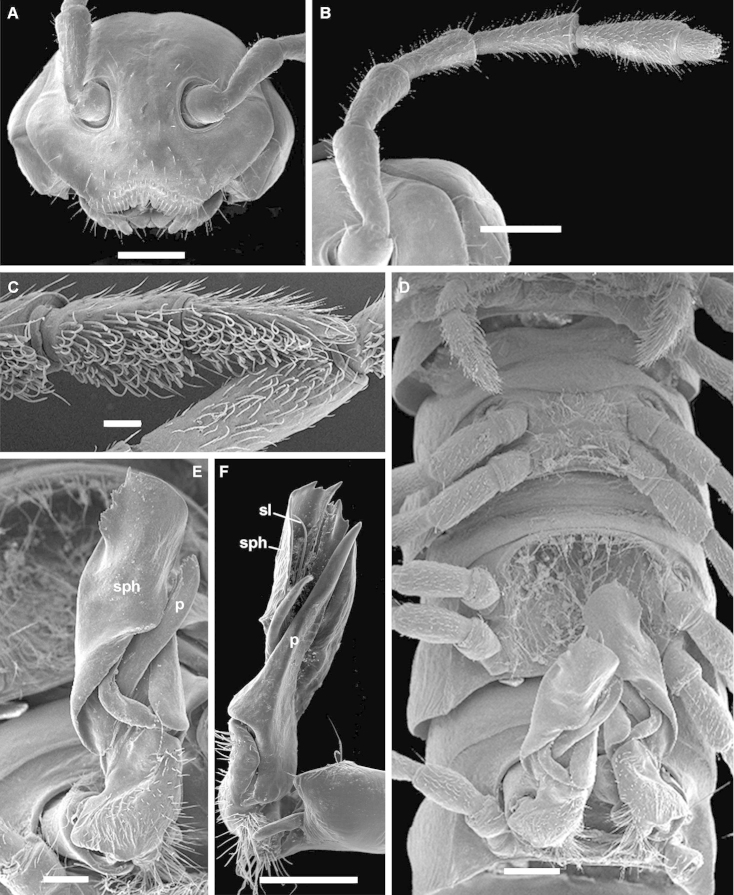
*Eviulisoma silvestre* (Carl, 1909), ♂ from Kakamega Forest, Kenya. **A** head, frontal view **B** antenna, frontal view **C** ventral brushes on tibia and tarsus, ventral view **D** body segments 4–7, ventral view **E, F** right gonopod, ventral and mesal views, respectively. Scale bars: 0.5 (**A, B, D, F**), 0.2 (**E**) & 0.1 mm (**C**). Designations in text.

This is only the second record of this species which has hitherto been known solely from Bakoba, S00°11', E37°28', Tanzania (Carl 1909). First described as a variety of *Eviulisoma fossiger* (Carl, 1909), it has since been treated (Hoffman 1953) as a species of full rank, recently very nicely revised and illustrated by Jeekel (2003) from type material. Even though the new samples, which are in rather poor condition, fully match Carl’s (1909) original description and Jeekel’s (2003) redescription, we provide additional illustrations (Fig. 11) to document the identity of this species. The shapes and proportions of the solenophore (**sph**), solenomere (**sl**) and postfemoral process (**p**) which has a conspicuous, parabasal, unciform branch (**h**) are quite characteristic. *Eviulisoma silvestre* appears to be very widely distributed, occurring not only in Tanzania, but also in Kenya (Map 1).

### Key to *Eviulisoma* species known from Kenya, based mainly on ♂ characters

**Table:** 

1	Sterna between ♂ legs 6 and 7 flattened, not excavate (Figs 1C, 2C). Paraterga 2 present, however small. Ngaia Forest (N 00°19', E 38°02')	**2**
–	Sterna between ♂ legs 6 and 7 deeply excavate and ledge-shaped for accommodation of gonopod tips (Figs 3C, 4H, I, 5C, 6E, F, 7C, 8C, 9C, 10C, 11D). Paraterga 2 sometimes totally absent	**4**
2	Sternal cones absent. Sternal lobe between ♂ coxae 4 large (Fig. 2C). Gonopod postfemoral process (**p**) large, phylloid, acuminate, but much shorter than a digitiform, suberect, apically rounded, lamellar solenophore (**sph**) (Fig. 2C–E)	***Eviulisoma ngaia* sp. n.**
–	Sternal cones present, starting from ♂ body segment 8. Sternal lobe between ♂ coxae 4 rather small, slightly concave (Fig. 1C). Gonopod postfemoral process (**p**) vestigial, solenophore (**sph**) longest and claw-shaped apically (**c**), with two characteristic teeth (**m** and **l**) in distal 1/3 (Fig. 1C–E)	***Eviulisoma ngaiaorum* sp. n.**
3	All ♂ telopodite segments distal to coxa or prefemur with ventral brushes. Epiproct with two distinct apical claws directed ventrad (Fig. 7b). Gonopods divergent, rather loose, with a complex, lamellar, apically unciform (**u**) solenophore (**sph**) partly sheathing a longer and flagelliform solenomere (**sl**); postfemoral process (**p**) very simple, strong and sickle-shaped (Fig. 7C–F)	***Eviulisoma kirimeri* sp. n.**
–	Only 2–3 last telopodite segments distal to coxa or prefemur in ♂ with ventral brushes. Epiproct with only inconspicuous apical papillae. Gonopods either held parallel to each other or somewhat convergent, always compact	**4**
4	Paraterga 2 wanting	**5**
–	Paraterga 2 at least traceable	**7**
5	Sternal cones totally absent. Hypoproct acute caudally. Gonopod postfemoral process longest, erect, digitiform, fringed at base on mesal face; both solenophore and solenomere only a little shorter, subequal in length, distal 1/3 of solenophore a subflagelliform branch	***Eviulisoma pallidum***
–	Sternal cones present at least between each caudal leg-pair per ♂ diplosegment following 7^th^. Hypoproct rounded caudally. Gonopod structure different, postfemoral process much longer than a similarly spiniform solenophore showing a fold for sheathing a likewise long solenomere in distal 1/3 extent	**6**
6	Sternal cones present only between each caudal leg-pair per ♂ diplosegment following 7^th^. Gonopod postfemoral process simple, not grooved longitudinally	***Eviulisoma jeanneli***
–	Sternal cones small, but present between both leg-pairs per ♂ diplosegment following 7^th^. Gonopod postfemoral process (**p**) more complex, grooved longitudinally, with a dorsal spinule in distal half (Fig. 10)	***Eviulisoma alluaudi***
7	Sternal lobe between ♂ coxae 4 missing (Fig. 3C). Gonopods (Figs 3C, 4H–L) very slender, with solenophore (**sph**), postfemoral process (**p**) and solenomere (**sl**) subequal in length. Sufficiently abundant samples revealing two distinct size morphs, with midbody widths being 1.5–2.7 or 2.0–3.7 mm	***Eviulisoma taitaorum* sp. n.**
–	Sternal lobe between ♂ coxae 4 usually present. Gonopods different. No distinct size morphs noted even in the syntopically occurring congener, *Eviulisoma taita* sp. n.	**8**
8	Hypoproct trapeziform, with a sharp tooth caudally. Sternal lobe between ♂ coxae 4 very small to missing. Sternal cones behind body segment 7 absent. Gonopod postfemoral process long and subspiniform, nearly as long as solenomere and a lamellar, fold-shaped solenophore, the latter showing a parabasal, unciform process about half as long as postfemoral process	***Eviulisoma silvestre***
–	Hypoproct broadly and regularly rounded caudally. Sternal lobe between ♂ coxae 4 always quite conspicuous. Sternal cones behind ♂ body segment 7 present. Gonopods different	**9**
9	Gonopods (Figs 5C, 6F–J) with a lamellar, slender, apically unciform and bidentate solenophore (**sph**) carrying a lateral tooth midway (**t**) and reaching about as long as a flagelliform solenomere (**sl**), both **sph** and **sl** being considerably higher than a rather simple, similarly slender, postfemoral process (**p**)	***Eviulisoma taita* sp. n.**
–	Gonopod solenophore (**sph**) complex, cup-shaped, lamellar, about as long as a flagelliform solenomere (**sl**), flanked medially by a long, subspiniform, postfemoral process (**p**) (Figs 8C, 9C–E)	***Eviulisoma kakamega* sp. n.**

## Conclusion

At least in Kenya, several places appear to support two *Eviulisoma* species, e.g. Ngaia Forest, Taita Hills and Kakamega Forest. Furthermore, one of the species from Taita Hills demonstrates remarkable size dimorphism, when adult males can vary in size by 1.5–2.0 times, and is parapatric with a second *Eviulisoma* species. We are not aware of anything similar among other Paradoxosomatidae, but some Odontopygidae, a purely Afrotropical family of Spirostreptida, also show surprisingly distinct size dimorphism (Didier VandenSpiegel, unpublished results). As noted above, this variability may be advantageous for the local populations in adverse ecological conditions, possibly allowing for selection of different life strategies.

Last but not least, even though *Eviulisoma* is already the largest paradoxosomatid genus in tropical Africa, at the moment counting 36 species or subspecies, there is little doubt that numerous further species will be discovered in the region.

## Supplementary Material

XML Treatment for Eviulisoma ngaia

XML Treatment for Eviulisoma ngaiaorum

XML Treatment for Eviulisoma taitaorum

XML Treatment for Eviulisoma taita

XML Treatment for Eviulisoma kirimeri

XML Treatment for Eviulisoma kakamega

XML Treatment for Eviulisoma alluaudi

XML Treatment for Eviulisoma silvestre
